# Drinking Water Quality and Health Risk Assessment in Rural Ghana: Evidence from North-East and North Gonja Districts in the Savannah Region

**DOI:** 10.3390/ijerph23060821

**Published:** 2026-06-22

**Authors:** Elvis Kichana, Solomon A. Minyila, Braimah Apambire, Collins Gbeti, Abukari Wumbei, Fati Alhassan

**Affiliations:** 1 World Vision Ghana, Accra, Ghana; ekichana@gmail.com (E.K.); sminyila@gmail.com (S.A.M.); 2 Center for International Water and Sustainability, Desert Research Institute, 2215 Raggio Pkwy, Reno, NV 89512, USA; 3 Drylands Research Institute, University for Development Studies, Tamale NL-1142-8658, Ghana; 4 Development Impact International, Accra P.O. Box AQ 156, Ghana

**Keywords:** drinking water quality, nitrate contamination, *Escherichia coli*, groundwater, rural Ghana, WASH, public health, SDG 6

## Abstract

**Highlights:**

**Public health relevance—How does this work relate to a public health issue?**

- Household drinking water shows widespread microbial contamination.
- Groundwater sources present emerging nitrate-related health risks.

**Public health significance—Why is this work of significance to public health?**

- Nearly half of the sources exceed the nitrate guideline in North Gonja.
- First integrated microbial, chemical, and health-risk assessment in the study area.

**Public health implications—What are the key implications or messages for practi-tioners, policy makers and/or researchers in public health?**

- Weak treatment and unsafe handling compromise water safety.
- Targeted chlorination and nitrate monitoring are urgently needed.

**Abstract:**

Background: Access to safe drinking water remains a critical public health concern in rural Ghana, particularly in climatically vulnerable and underserved settings. This study assessed the microbiological and chemical quality of drinking water and evaluated nitrate-related health risks in the North Gonja and North-East Gonja Districts of the Savannah Region. Methods: A cross-sectional study was conducted between January and March 2025. A total of 460 water samples were collected from groundwater sources and household storage containers. Microbial analyses targeted total coliforms and *Escherichia coli*. Physicochemical and chemical parameters included nitrate-nitrogen, pH, residual chlorine, major ions, and trace metals. Data was analyzed using descriptive statistics, chi-square tests, spatial interpolation, and non-carcinogenic health risk assessment based on the hazard quotient (HQ) approach. Results: Widespread microbial contamination was observed, with 91.5% of household water samples positive for total coliforms and 46.6% for *E. coli*. Contamination of source water was significantly higher in North Gonja than in North-East Gonja. Overall, 49.1% (*n* = 55) of groundwater sources exceeded the World Health Organization guideline value for nitrate-nitrogen, with exceedances predominantly occurring in North Gonja. Additionally, 67.0% (*n* = 75) of samples were outside the acceptable pH range (6.5–8.5), including 74 samples below 6.5 and one above 8.5. Residual chlorine was not detected in any of the samples. Health risk assessment indicated potential non-carcinogenic risks associated with nitrate exposure, particularly among infants and children. Conclusions: The study demonstrates significant microbial contamination and nitrate-related health risks in the study area, particularly in North Gonja. Interventions such as improved source protection, routine water quality monitoring, chlorination, household water treatment, and implementation of Water Safety Plans are recommended to enhance drinking water safety and reduce associated public health risks.

## 1. Introduction

Access to safe drinking water is fundamental to public health, economic development, and social well-being. Despite progress toward Sustainable Development Goal (SDG) 6, more than 2 billion people globally still lack access to safely managed drinking water services, particularly in low- and middle-income countries [1,2]. Consumption of unsafe water contributes to diarrhoeal diseases, environmental enteropathy, malnutrition, and elevated childhood mortality, with the greatest burden observed in sub-Saharan Africa [2,3]. Microbial contamination, especially from Escherichia coli, remains the primary health risk in rural water supplies [4,5]. In addition, chemical contaminants such as nitrate, arsenic, and fluoride pose significant long-term health risks, even when adverse effects are not immediately evident [6,7,8].

Recent evidence indicates that access to an “improved” water source does not necessarily ensure water safety [5]. Systematic reviews show that water quality often deteriorates between the source and the point of use, largely due to unsafe handling, transport, and storage practices [5,9]. While some studies emphasize infrastructure expansion as a key strategy for improving water safety, others highlight the importance of source protection, household-level treatment, and routine monitoring [10,11]. This divergence reflects an ongoing debate within water, sanitation, and hygiene (WASH) research regarding the extent to which infrastructure alone can improve health outcomes.

In Ghana, although access to drinking water has increased, substantial water quality challenges persist, particularly in rural and vulnerable communities [12,13]. Studies from northern Ghana have reported nitrate contamination of groundwater linked to agricultural activities and inadequate sanitation, as well as microbial contamination resulting from poor wellhead protection and seasonal flooding [14,15,16]. However, most previous studies have examined microbiological and chemical risks in isolation, limiting comprehensive understanding of cumulative exposure and contamination pathways.

The Savannah Region of Ghana is characterized by a semi-arid climate, hydrogeologically vulnerable Voltaian aquifers, seasonal flooding, and limited routine water quality monitoring. These environmental and institutional conditions are likely to exacerbate both microbial and chemical contamination risks [17,18]. Groundwater degradation in Ghana and across sub-Saharan Africa has been associated with anthropogenic pressures, including inadequate sanitation, agricultural intensification, and hydrogeological vulnerability. In Ghana, nitrate contamination has been linked to sewage disposal, manure management, septic system leakage, and fertilizer application, contributing to public health concerns in both the Lower and White Volta River Basins [14,19]. More broadly, elevated nitrate concentrations are widely recognized as indicators of groundwater pollution, particularly in shallow aquifers affected by agriculture, domestic waste disposal, urbanization, and insufficient source protection [18]. These challenges are especially pronounced in rural communities that rely on groundwater and have limited water quality surveillance and risk-based management systems. Despite growing evidence of groundwater contamination, few studies in the Savannah Region have simultaneously assessed microbiological and chemical water quality, household-level contamination pathways, and associated health risks.

At present, there is a lack of district-level studies that concurrently examine microbiological and chemical risks at the point of collection and at the point of use. Beyond compliance with drinking water standards, quantitative health risk assessment offers a robust framework for evaluating potential human health impacts of chemical contaminants. The United States Environmental Protection Agency (USEPA) recommends non-carcinogenic risk assessment approaches based on chronic daily intake (CDI) and hazard quotient (HQ) [20,21]. These models integrate contaminant concentrations, water ingestion rates, and body weight to estimate exposure, which is then compared with established reference doses (RfD). For nitrate-nitrogen, the RfD (1.6 mg/kg/day) is based on the risk of methemoglobinemia, particularly among infants, and serves as a widely accepted benchmark for non-carcinogenic risk evaluation [6,22]. Unlike simple guideline comparisons, this approach quantifies exposure levels across population groups, providing a stronger basis for risk-informed public health decision-making. Such approaches are particularly relevant in rural groundwater systems, where contaminant levels exhibit spatial variability and vulnerable populations may face disproportionate exposure.

This study addresses a critical evidence gap at the district level by jointly assessing microbiological contamination, nitrate pollution, pH variability, residual chlorine status, and post-collection contamination at the household level in groundwater-based drinking water systems. By integrating source- and household-level data with spatial and statistical analyses, the study provides context-specific insights into contamination pathways and exposure risks in rural northern Ghana. The findings are intended to inform Water Safety Planning, strengthen district-level surveillance systems, and support risk-based WASH interventions in the Savannah Region.

## 2. Materials and Methods

### 2.1. Study Area

This study was conducted in the North-East Gonja and North Gonja Districts, located in the Savannah Region of northern Ghana. The Savannah Region lies within the Guinea-Savannah agro-ecological zone, characterised by prolonged dry seasons, erratic rainfall, and recurring water scarcity. Geologically, the two districts are underlain by the Voltaian formation, which has limited groundwater occurrence. These factors, combined with poverty and limited infrastructure, pose significant challenges to water access and safety.

#### 2.1.1. North-East Gonja District

**Figure 1:**
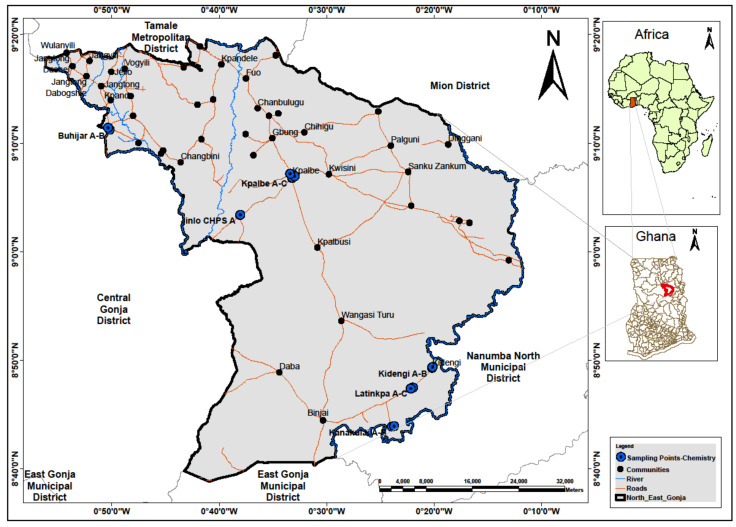
Spatial Map/Sampling points of North-East Gonja District.

The North-East Gonja District (Figure 1), located within the Savannah Region, borders East Gonja Municipality to the south, Yendi Municipality to the north-east, and Tamale Metropolis to the north-west. Its administrative capital, Kpalbe (~9.115° N, −0.553° W), anchors a predominantly rural landscape characterized by dispersed settlements that depend on boreholes, hand-dug wells, and small-scale mechanised water systems. The population relies heavily on subsistence farming and seasonal fishing along the White Volta River and ephemeral streams. Low economic resilience and limited infrastructure make year-round access to safe water a persistent challenge, especially during dry seasons.

The district lies within the Voltaian Basin, where variable geochemical conditions shape groundwater quality. Fluoride and iron levels occasionally exceed recommended limits, while historical records show that microbial contamination poses the greatest threat to public health. Only a limited number of functional boreholes and water systems serve the area, and many of them lack systematic maintenance and regular monitoring. These conditions highlight the urgent need to strengthen water quality management and implement targeted WASH interventions.

#### 2.1.2. North Gonja District

**Figure 2:**
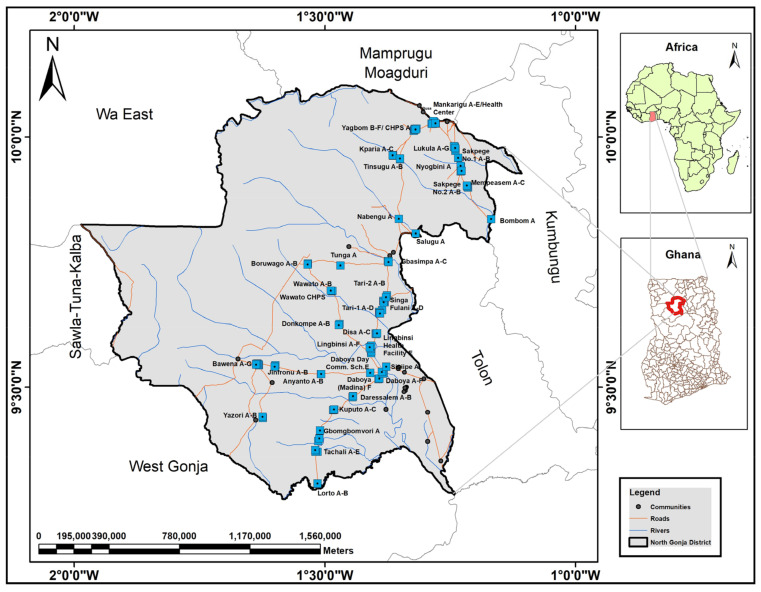
Spatial Map/Sampling points of North Gonja District.

North Gonja District (Figure 2), located west of Northeast Gonja, borders West Gonja Municipality, Kumbungu District, and Savelugu District. Its capital, Daboya (~9.566° N, −0.983° W), lies along the White Volta River and serves as a critical service hub. Communities such as Mempeasem, Lingbinsi, and Lukula lie in low-lying floodplains, making them highly vulnerable to seasonal flooding. Seasonal floods frequently contaminate shallow aquifers and surface water sources, exposing the population to significant public health risks. Although development partners have introduced mechanised water systems, local operators rarely carry out consistent disinfection or routine water quality testing.

### 2.2. Study Design and Sampling Strategy

A cross-sectional study was conducted over a ten-week period, from 20 January to 20 March 2025, in selected communities within the North Gonja and North-East Gonja Districts of the Savannah Region, Ghana. The objective was to assess microbiological and chemical water quality at two levels: water sources (boreholes and limited mechanized systems) and household storage containers. A multistage sampling design, incorporating both probability and nonprobability techniques, was used to ensure representation of different geographical areas and water source types. During data collection, 145 functional water systems were identified in the two districts, from which 112 boreholes were selected. The estimated household population in the selected study communities was approximately 15,210. The Krejcie and Morgan, 1970 [23] sample size determination method was used to obtain a representative sample of 357 households, which was proportionally allocated among the communities based on population size. This method ensured sufficient statistical representation and operational feasibility during field implementation. Household selection involved geographic stratification into four zones (north, south, east, and west), followed by random sampling without replacement within each zone. Boreholes serving public facilities, such as schools and health centers, were purposively included. In communities with multiple systems, simple random sampling was conducted using borehole maps to achieve spatial balance.

A total of 460 water samples were collected and analyzed: 348 for microbiological testing (112 source and 236 household samples) and 112 for chemical analysis. Of these, 14 source and 27 household samples originated from the North-East Gonja District, while 98 source and 209 household samples were from the North Gonja District. All sampling and analytical procedures followed the Standard Methods for the Examination of Water and Wastewater (24th ed.) [24] and were conducted in the Regional Water Quality Laboratory of World Vision International (Ghana), ensuring traceability, quality assurance, and compliance with international standards.

**Figure 3:**
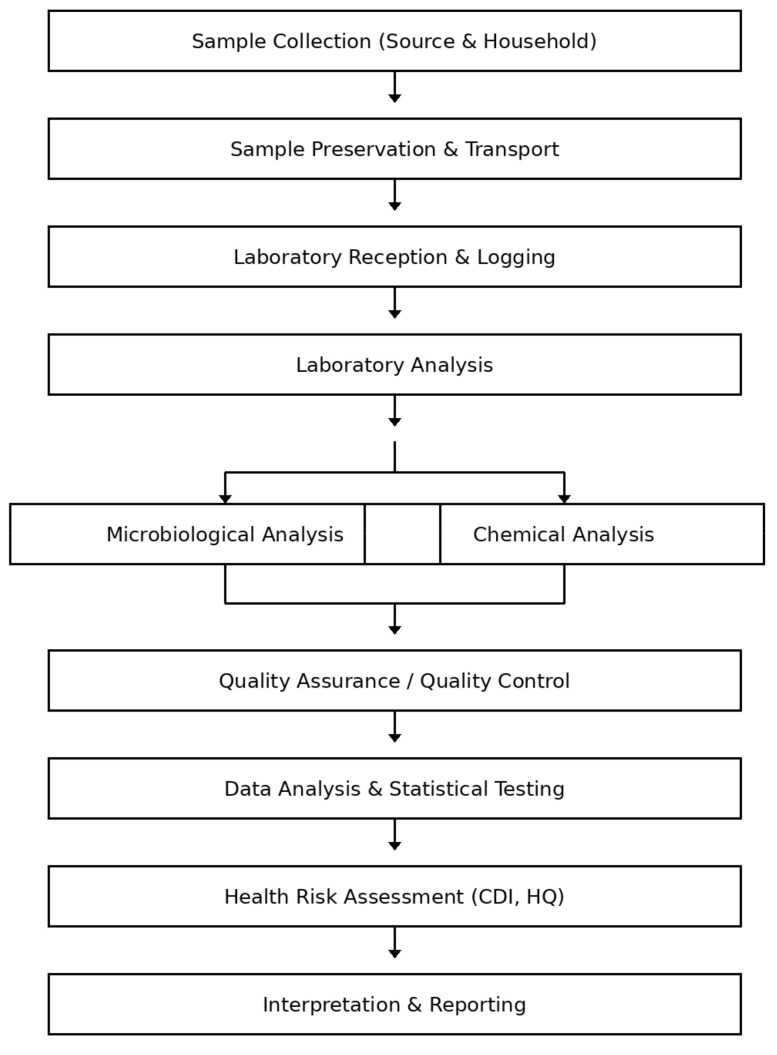
Schematic representation of the methodological workflow for water sample collection, analytical procedures, and data processing.

Figure 3 presents a flow diagram outlining the sequence of sample collection, handling, laboratory analysis, quality assurance and quality control procedures, and data analysis, providing a clear and concise overview of the methodological approach.

### 2.3. Sample Collection and Transportation

Water samples for microbiological analysis were collected in duplicate using aseptic techniques to prevent contamination from external sources. IDEXX 100 mL sterile polypropylene bottles containing sodium thiosulfate (Na_2_S_2_O_3_) (IDEXX Laboratories, Inc., Westbrook, ME, USA) were used for residual chlorine neutralization and sample collection.

For chemical analysis, duplicate water samples were collected in pre-rinsed 500 mL high-density polyethylene bottles. The first sample in each duplicate set was acidified to approximately pH 2 with trace-grade nitric acid (HNO_3_) (Thermo Fisher Scientific, Waltham, MA, USA) for preservation and then used for trace-metal and cation analyses. The second duplicate, which was not acidified, was used for physical parameters and chemical ion analysis. All samples were labeled, logged, and transported in cool, insulated boxes maintained below 10 °C. Sample holding times for microbiological analysis did not exceed 6 h.

### 2.4. Analytical Methods

Laboratory analyses were performed at the World Vision Regional Water Quality Laboratory (RWQL) in Tamale, in accordance with internal quality control procedures aligned with ISO/IEC 17025:2017 [25].

#### 2.4.1. Microbiological Analysis

Microbial contamination in water samples was assessed using the membrane filtration technique with Brilliance *E. coli*/Coliform Selective Agar (Oxoid Ltd., Basingstoke, Hampshire, UK). This chromogenic medium enables simultaneous detection and enumeration of *E. coli* and total coliforms through differential color development.

##### Principle (Brilliance *E. coli*/Coliform Selective Agar)

Brilliance *E. coli*/Coliform Selective Agar is a chromogenic medium that detects β-D-glucuronidase (GUD) and β-D-galactosidase (GAL) activities to differentiate *Escherichia coli* from other coliforms. The medium contains two chromogenic substrates: X-Glu for GUD and Rose-Gal for GAL. *E. coli* typically expresses both enzymes and hydrolyzes both substrates, producing purple colonies, while other coliforms express only GAL and appear pink. Sodium lauryl sulfate inhibits Gram-positive flora, and the presence of tryptophan allows for same-day indole confirmation of *E. coli* if necessary.

##### Procedure

A 100 mL aliquot of water was aseptically filtered through a sterile 0.45 μm cellulose nitrate membrane filter using a vacuum filtration unit. The membrane was then transferred onto prepared Brilliance *E. coli*/Coliform Agar plates and incubated at 36 ± 0.5 °C for 24 ± 2 h. After incubation, colonies were enumerated and results reported as Colony Forming Units per 100 mL (CFU/100 mL). Results were interpreted according to WHO [1] drinking water quality guidelines, which require the absence of detectable *E. coli* per 100 mL for compliance.

#### 2.4.2. Chemical Analysis

Water quality parameters were analyzed using standard procedures described in the Standard Methods for the Examination of Water and Wastewater [24] and complementary U.S. EPA methods. The selected parameters included general physicochemical properties (pH, electrical conductivity, turbidity), major ions (nitrate, fluoride, chloride, calcium, potassium, sodium, magnesium, carbonates, and bicarbonates), and trace metals, which are key indicators of drinking water quality. The analytical techniques and their reference methods are summarized in Table 1 below.

#### 2.4.3. Quality Assurance and Quality Control (QA/QC)

A thorough quality assurance and quality control (QA/QC) protocol was used to maintain reliable analysis and high data quality. Calibration verification standards were analyzed at the beginning and end of each analytical batch. Calibration curves were accepted only when coefficients of determination (R^2^) were ≥0.995. Duplicate samples, laboratory control samples, matrix spikes, and certified reference materials were evaluated in accordance with laboratory quality control procedures aligned with ISO/IEC 17025:2017 requirements to verify analytical precision, accuracy, and traceability. These steps together helped ensure the results were accurate, precise, and reproducible.

Microbiological analyses were performed in accordance with established laboratory biosafety procedures and internal quality management protocols consistent with ISO/IEC 17025:2017. Biohazardous materials, such as used membrane filters, culture plates, contaminated consumables, and residual water samples, were treated appropriately prior to disposal. Decontamination was achieved through autoclaving or chemical disinfection, depending on material type, to ensure complete inactivation of microbial contaminants. Laboratory work surfaces and equipment were disinfected before and after each analytical session. Personnel followed standard biosafety practices, including the use of personal protective equipment (laboratory coats, gloves) and strict hand hygiene. These procedures maintained both analyst safety and the integrity of microbiological results.

### 2.5. Water Quality Parameter Selection

Water quality parameters were selected based on their significance for drinking-water safety, public health risk, local hydrogeological context, and operational monitoring needs. Microbiological indicators, specifically *Escherichia coli* and total coliforms, served as primary markers of fecal contamination and immediate health risk, consistent with World Health Organization (WHO) guidelines for drinking-water quality [1]. Physicochemical and chemical parameters, including nitrate-nitrogen (NO_3_-N), fluoride, chloride, sulfate, pH, turbidity, electrical conductivity (EC), total dissolved solids (TDS), and selected trace metals, were chosen due to their documented presence in groundwater systems, relevance to acute and chronic health risks as well as aesthetic effects that could cause rejection, and inclusion in WHO and Ghana drinking-water standards (GS 175:2024) [1,26]. Residual chlorine was incorporated as an operational parameter to evaluate disinfection efficacy and the continuity of the water safety chain, particularly in small and community-managed water systems. This risk-based selection strategy addresses both immediate microbiological hazards and long-term chemical risks.

### 2.6. Residual Chlorine Testing

Residual chlorine in all drinking water samples was measured using HACH Residual Chlorine Test Strips (Model 2745050) (HACH Company, Loveland, CO, USA) to assess disinfection effectiveness. The strips provided rapid, semi-quantitative measurements of free chlorine by reacting with N, N-diethyl-phenylenediamine (DPD) to generate a colorimetric signal. Each strip was briefly dipped into a freshwater sample and held flat for about 10 s to allow the color to develop. The color was then compared immediately with the manufacturer’s chart to determine the free chlorine level, typically between 0 and 10 mg/L. Results were recorded in mg/L and compared to the WHO [1] minimum guideline of 0.2 mg/L for effective microbial control. Samples with free residual chlorine levels below 0.2 mg/L were considered improperly disinfected.

### 2.7. Non-Carcinogenic Health Risk Assessment for Nitrate-Nitrogen

The non-carcinogenic health risk associated with nitrate-nitrogen (NO_3_-N) exposure through drinking water ingestion was estimated using the chronic daily intake (CDI) and hazard quotient (HQ) methods, as recommended in established human health risk assessment frameworks [20,21]. The assessment was performed separately for North Gonja and North-East Gonja Districts to facilitate district-specific comparisons of nitrate-related exposure risk. Chronic daily intake (CDI) was calculated according to Equation (1):

CDI = (C × IR)/BW(1)

In this equation, CDI represents the chronic daily intake of nitrate-nitrogen through drinking water (mg/kg/day), C is the measured concentration of NO_3_-N in drinking water (mg/L), IR denotes the daily drinking water ingestion rate (L/day), and BW is body weight (kg). Exposure assumptions for ingestion rate and body weight were derived from standard USEPA exposure factor guidance [21] because site-specific intake and body weight data were unavailable.

The hazard quotient (HQ) was determined using Equation (2):

HQ = CDI/RfD(2)

where HQ denotes the non-carcinogenic hazard quotient.

RfD is the oral reference dose for nitrate-nitrogen.

An RfD of 1.6 mg NO_3_-N/kg/day was used, consistent with the USEPA Integrated Risk Information System (IRIS) value for nitrate [22]. An HQ value of 1 or less suggests that adverse non-carcinogenic health effects are unlikely, while HQ values greater than 1 indicate a potential health concern and the need for intervention [20].

CDI and HQ values were estimated using the mean, 95th percentile, and maximum NO_3_-N concentrations for each district to represent typical, upper-bound, and worst-case exposure scenarios. This approach offers a more health-relevant interpretation of nitrate exposure than guideline-based compliance assessment alone [6].

### 2.8. Data Analysis

Laboratory data were analyzed using quantitative statistical methods in Microsoft Excel (version 2021) (Microsoft Corporation, Redmond, WA, USA). The analysis focused on the following areas:

i. Descriptive statistics for each parameter.
ii. Chi-Square Test/Analysis.
iii. Proportional occurrence rates of microbial contamination.
iv. Comparative assessment between districts and between source and household samples.
v. Conformance analysis against WHO guideline values [1].

## 3. Results

### 3.1. Microbiological Quality of Source Water

**Figure 4:**
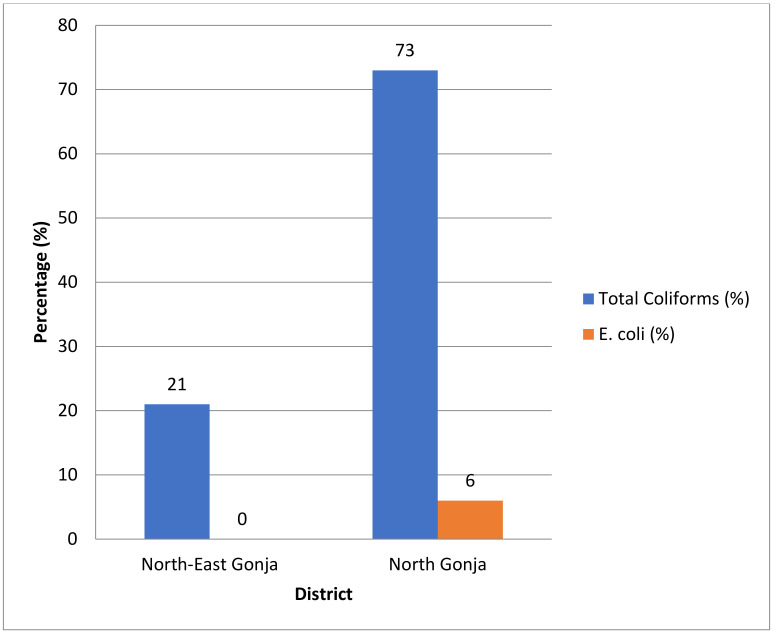
Bar graph showing the microbiological Quality of Source Drinking Water.

Among the 112 source water samples analyzed, total coliforms were detected in 75 (67.0%), and *Escherichia coli* (*E. coli*) was detected in 6 (5.4%). The degree of microbial contamination differed significantly between the two study districts. In North Gonja District, 72 of 98 samples (73.5%) tested positive for total coliforms, and 6 samples (6.1%) were positive for *E. coli*. In contrast, North-East Gonja District showed considerably lower contamination, with only 3 of 14 samples (21.4%) testing positive for total coliforms and no *E. coli* detections (0.0%). These results demonstrate a marked spatial disparity in the microbial quality of source water between the two districts, as shown in Figure 4.

### 3.2. Microbiological Quality of Household Water

**Figure 5:**
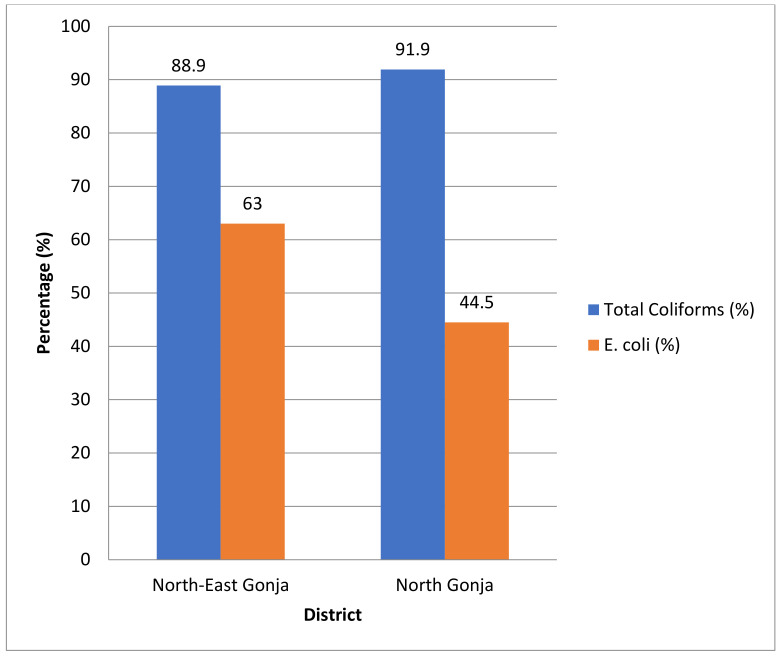
A bar graph showing the microbiological quality of household drinking water.

Of the 236 household water samples tested, 216 (91.5%) were positive for total coliforms, and 110 (46.6%) contained *Escherichia coli* (*E. coli*). The levels of microbial contamination differed between the two districts. In North Gonja District, 91.9% of samples tested positive for total coliforms, and 44.5% tested positive for *E. coli*. In North-East Gonja District, 88.9% of samples tested positive for total coliforms, and 63.0% tested positive for *E. coli*. Figure 5 compares contamination rates across districts.

### 3.3. Assessment of Chemical Water Quality

A total of 112 groundwater source samples were analyzed for physicochemical and chemical parameters to evaluate compliance with World Health Organization (WHO) and Ghanaian drinking-water standards [1,26]. Table 2 presents the descriptive statistics for all measured parameters.

Most parameters, including total dissolved solids (TDS), conductivity, fluoride, chloride, sulfate, and most trace metals, were within guideline limits. In contrast, nitrate-nitrogen, pH, and residual chlorine were identified as primary concerns. Nitrate-nitrogen concentrations ranged from 0.35 to 72.00 mg/L, with 49.1% of samples exceeding the WHO guideline of 11.3 mg/L, indicating significant localized contamination. pH ranged from 4.40 to 8.52, with 67.0% of samples outside the acceptable range (6.50–8.50), indicating widespread acidity.

Residual chlorine was undetectable in all samples. Nitrate-nitrogen and pH showed the greatest departures from guideline values. A comprehensive descriptive statistical analysis of physicochemical and chemical parameters is presented in Table 2, summarizing central tendency, variability, and compliance with WHO and Ghana drinking-water guideline values. For clarity, the physicochemical and chemical water quality results are organized into three categories: physical parameters (Table 2A), major ions (Table 2B), and trace metals (Table 2C).

### 3.4. Hydrogeochemical and Spatial Distribution of Nitrate (N) and pH in Groundwater in North Gonja and North-East Gonja Districts

**Figure 6:**
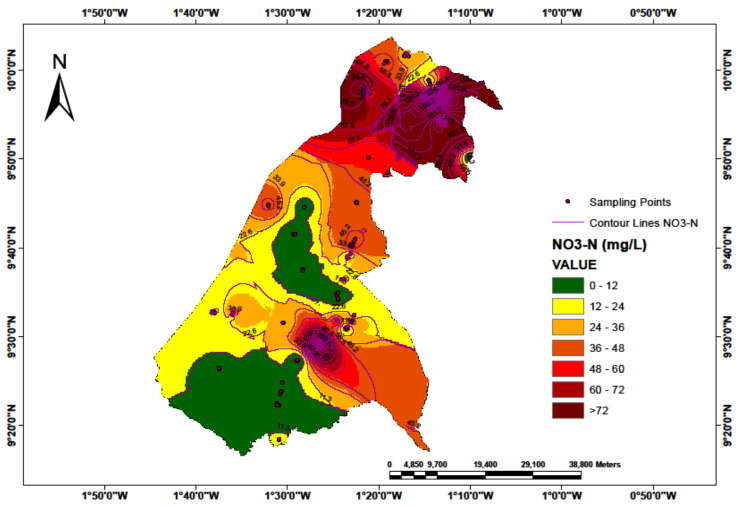
Nitrate(N) (North Gonja).

**Figure 7:**
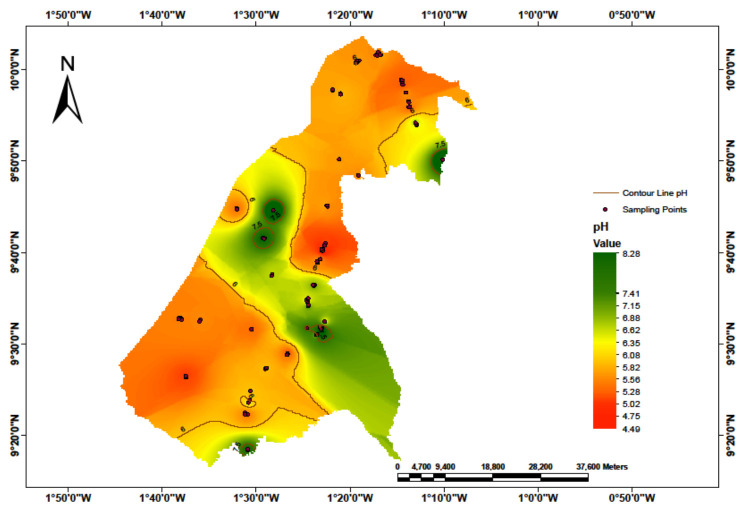
pH (North Gonja).

**Figure 8:**
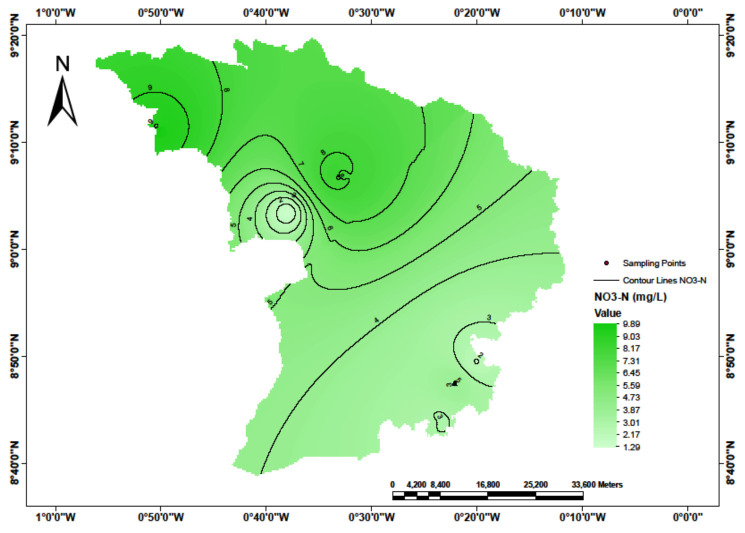
Nitrate (N) (North-East Gonja).

**Figure 9:**
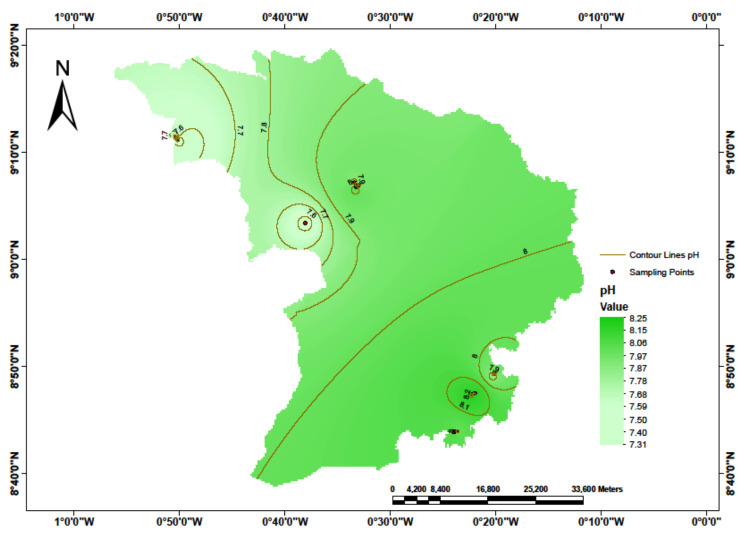
pH (North-East Gonja).

Figure 6, Figure 7, Figure 8 and Figure 9 display spatial contour maps of nitrate-nitrogen (NO_3_-N) and pH distributions across the North Gonja and North-East Gonja Districts in the Savannah Region of Ghana. These maps were generated using the Inverse Distance Weighting (IDW) interpolation method in ArcGIS 10.8 (Esri, Redlands, CA, USA) to visualize spatial variability and identify potential contamination hotspots.

In North Gonja, NO_3_-N concentrations ranged from 0 to over 72 mg/L, while in North-East Gonja, concentrations were significantly lower, ranging from 1.29 to 9.89 mg/L. Groundwater pH in North Gonja exhibited substantial variability (4.49–8.28), indicating conditions ranging from acidic to slightly alkaline. In contrast, North-East Gonja showed relatively stable pH values, ranging from neutral to slightly alkaline (7.31–8.25).

The contour maps visually demonstrate hydrogeochemical variations both within and between the two districts, reflecting differences in land use, recharge dynamics, and aquifer geochemistry. Collectively, these maps support the assessment of potential anthropogenic influences, nitrification-induced acidification, and the buffering capacity of the underlying geological formations.

### 3.5. Nitrate-Nitrogen Health Risk Assessment

A non-carcinogenic health risk assessment was conducted to evaluate the public health implications of nitrate contamination, utilizing the hazard quotient (HQ) approach based on chronic daily intake (CDI). The HQ was calculated as follows:

HQ = (C × IR/BW)/RfD

In this equation, C represents the nitrate-nitrogen concentration (mg/L), IR denotes the daily ingestion rate (L/day), BW is body weight (kg), and RfD is the reference dose. An RfD of 1.6 mg NO_3_-N/kg/day was applied in accordance with USEPA guidelines. Due to the lack of site-specific exposure data, water ingestion rates and body weights for different population groups were based on standard USEPA exposure factors widely used in drinking-water health risk assessments. These assumptions serve as accepted default values for estimating population-level exposure in environmental health studies. The assessment included infants, children, adults, and pregnant women, and utilized mean, 95th percentile, and maximum nitrate-nitrogen concentrations to represent typical and upper-bound exposure scenarios.

For infants exposed to a mean nitrate-nitrogen concentration of 23.28 mg/L, the chronic daily intake (CDI) was calculated as follows:

CDI = (23.28 × 0.78)/10 = 1.82 mg/kg/day.

The corresponding hazard quotient (HQ) was 1.14. Similar calculations were performed for additional population groups and exposure scenarios, as summarized in Table 3. An HQ greater than 1 indicates a potential non-carcinogenic health risk from nitrate exposure. All calculations utilized United States Environmental Protection Agency (USEPA) exposure assumptions and a reference dose (RfD) of 1.6 mg NO_3_-N/kg/day.

The results indicate that exposure to nitrate-nitrogen poses a potential non-carcinogenic health risk, particularly for infants and children. At the mean concentration, the hazard quotient (HQ) exceeded 1 for infants, while the HQ for children was near the risk threshold. At the 95th percentile and maximum concentrations, HQ values for infants, children, adults, and pregnant women either exceeded or approached the risk threshold, suggesting heightened concern under upper-bound exposure scenarios. Infants consistently exhibited the highest HQ values, attributable to greater water intake per body weight and increased physiological vulnerability. Even at the World Health Organization (WHO) guideline value, HQ values for infants were close to the threshold, underscoring the need for precautionary management. These findings suggest that nitrate contamination represents not only a regulatory compliance issue but also a significant public health risk, particularly for vulnerable population groups in the study area.

### 3.6. Chi-Square Analysis of Chemical Water Quality

A Chi-Square test (Table 4) was performed to assess whether exceedances of key chemical parameters were significantly associated with district location, specifically comparing North Gonja and North-East Gonja. The analysis included all source water samples collected from each district (North Gonja: 98; North-East Gonja: 14).

## 4. Discussion

This study evaluated the microbiological and chemical quality of drinking water in two resource-constrained districts, North-East Gonja and North Gonja, within the Guinea savanna ecological zone of the Savannah Region, Ghana. Although both districts share similar ecological and climatic characteristics, variations in hydrogeological conditions, land use practices, and levels of water-source protection contribute to spatial differences in drinking-water quality. The findings revealed clear disparities in water safety between the two districts, characterized by substantial post-collection household-level microbial contamination and elevated chemical pollution risks, particularly in North Gonja District. These results have important implications for public health, WASH policy, and the design of sustainable water supply interventions in rural Northern Ghana.

### 4.1. Disparities in Source Water Quality

The results reveal substantial disparities in source water quality between the two districts, with North Gonja demonstrating significantly higher microbial contamination than North-East Gonja. In North Gonja, 73.5% of source samples tested positive for total coliforms and 6.1% for *Escherichia coli*, while the corresponding values in North-East Gonja were 21.4% and 0.0%, respectively. These results suggest that groundwater sources in North Gonja are more susceptible to faecal contamination, likely attributable to a combination of hydrogeological and environmental factors. Comparable patterns have been documented in Ghana and other sub-Saharan African regions, where boreholes situated in low-lying or flood-prone areas are more prone to contamination due to shallow aquifer systems, inadequate drainage, and insufficient wellhead protection [16,27].

The absence of residual chlorine in all sampled water systems suggests deficiencies in water treatment and operational management. Residual chlorine serves as a critical barrier to microbial contamination, especially in rural water supply systems with limited infrastructure and monitoring capacity. The lack of detectable residual chlorine indicates that routine disinfection is either not implemented or not maintained, thereby increasing the risk of microbial persistence and regrowth throughout the distribution and storage chain. This observation aligns with previous studies in Ghana and similar rural contexts, which have found that small, community-managed water systems often lack consistent chlorination due to logistical challenges, limited technical expertise, and weak supply chains [28,29]. Although the Ghana National Water Policy requires the disinfection of public water systems, implementation and enforcement remain difficult in decentralized rural systems, particularly in northern regions [11].

### 4.2. Household-Level Contamination and Post-Source Risk Amplification

A key finding of this study was the widespread microbial contamination observed in household-stored water. A total of 91.5% of household water samples tested positive for total coliforms, and 46.6% tested positive for *Escherichia coli*. These proportions far exceed the World Health Organization’s guideline value of 0 cfu/100 mL for both indicators, signifying a high level of faecal contamination and post-collection deterioration of microbiological water quality.

The observed increase in microbial contamination from source water to household water indicates a decline in water quality following collection. Potential contamination pathways include unsafe transport methods, uncovered storage containers, use of utensils, insufficient household water treatment, and insufficient residual disinfectant levels [30].

These findings highlight a failure to maintain the water safety continuum from source to consumption and emphasize the need to integrate safe water-handling, storage, and residual disinfection into rural water supply management.

This pattern is consistent with observations by Wright et al. [9], who reported that water quality frequently deteriorates between the source and the point of use due to unsafe storage, dipping, and hand contamination.

In North-East Gonja, although the source water was microbiologically safe, 63.0% of household water samples tested positive for *E. coli*. This confirms the critical breakdown in the water safety continuum and supports findings from Clasen et al. [10], who demonstrated that effective household water management is essential, even in areas with improved water sources.

These contamination rates indicate not only technological deficiencies but also behavioural and social barriers, such as limited hygiene education, lack of covered containers, and prevailing cultural norms regarding water handling. This underscores the need for household-level interventions alongside infrastructure improvements.

### 4.3. Hydrogeochemical and Spatial Distribution of Nitrate–Nitrogen (NO_3_–N) and pH

The spatial contour maps of nitrate as nitrogen (NO_3_–N) and pH (Figure 6, Figure 7, Figure 8 and Figure 9) reveal distinct hydrogeochemical patterns between North Gonja and North-East Gonja Districts, reflecting differences in anthropogenic influence and aquifer geochemistry. In North Gonja District, NO_3_–N concentrations were highly variable, ranging from 0 mg/L to 72 mg/L or more, well above the WHO guideline value of 11.3 mg/L for safe drinking water. Elevated nitrate zones were predominantly found in the northern and northeastern sections of the district, where population density and agricultural or livestock activities are relatively higher (Daboya and Mankarigu). The spatial coincidence of these high-nitrate areas with acidic to slightly acidic pH values (4.49–6.5) suggests that nitrification of organic and ammonium-based nitrogen is a key process driving groundwater acidification. During nitrification, microbial oxidation of ammonium produces nitrate and releases hydrogen ions, lowering groundwater pH as shown in the reaction:

NH_4_^+^ + 2O_2_ → NO_3_^−^ + 2H^+^ + H_2_O.

Given the low carbonate buffering capacity typical of the lateritic aquifers in the Savannah Region, the generated acidity is not neutralized, resulting in the observed inverse spatial relationship between NO_3_–N and pH. These findings are consistent with earlier studies in northern Ghana and the Sahel, where acidic, nitrate-rich groundwater has been linked to shallow, oxidized aquifers under anthropogenic nitrogen loading [31,32,33].

The North-East Gonja District showed uniformly low nitrate concentrations (1.29–9.89 mg/L) and near-neutral to slightly alkaline pH (7.31–8.25), suggesting minimal nitrogen contamination and a stable geochemical environment. The weak spatial correspondence between pH and nitrate in this district indicates limited nitrification. The observed pattern is typical of less-impacted groundwater systems, in which natural recharge and lithological composition maintain chemical equilibrium.

A comparison between the two districts highlights clear differences in the influence of anthropogenic activities and in the aquifer’s buffering capacity on groundwater chemistry. In North Gonja District, elevated nitrate concentrations are spatially concentrated in densely populated areas, particularly around Daboya and Mankarigu, as shown by the nitrate contour maps. This spatial pattern indicates anthropogenic nitrate contamination and is accompanied by lower pH values, suggesting greater susceptibility to groundwater acidification. In contrast, the North-East Gonja District is characterized by generally lower nitrate concentrations and relatively stable pH, suggesting more pristine aquifer conditions and stronger geochemical buffering. Overall, an inverse relationship between nitrate and pH is evident, with a strong correlation observed in North Gonja and a weaker relationship in North-East Gonja. These findings indicate that nitrification-driven acidification, coupled with limited buffering capacity, plays a central role in degrading groundwater quality in the northern savannah. Similar spatial and geochemical patterns have been reported in the Afram Plains [27] and other groundwater systems in northern Ghana [32,33], supporting the geochemical plausibility of these results.

The observed acidic groundwater conditions may also enhance the mobilization of certain metals, including iron, manganese, and lead, by increasing their solubility at low pH, as established in the WHO Guidelines for Drinking-water Quality and hydrogeochemical studies on pH-dependent metal mobility [1,34].

### 4.4. Chronic and Geogenic Risks of Chemical Contaminants

The chemical assessment revealed high nitrate concentrations in 55 source samples (49.1%), all of which were from North Gonja. Nitrate values exceeded the WHO’s maximum recommended limit of 11.3 mg/L. Chronic exposure to nitrate-contaminated water is linked to methemoglobinemia in infants and potential carcinogenic effects [6]. These findings are consistent with hydrogeological studies in the Voltaian Basin, which have previously linked nitrate elevation to agricultural runoff, unlined latrines, and shallow aquifers [14,15].

### 4.5. Chi-Square Test/Analysis

The significant association between nitrate exceedances and acidic pH in North Gonja indicates spatial clustering of chemical water-quality challenges in the district. This pattern reflects findings from studies in the Lower Volta Basin and other parts of Northern Ghana, which link elevated nitrate levels and low pH to floodplain hydrogeology, agricultural runoff, and inadequate sanitation infrastructure. Our findings, which show that nearly half of the groundwater sources in North Gonja exceeded the WHO guideline value for nitrate, point to a combined influence of geogenic processes and anthropogenic inputs on groundwater chemistry. Such elevated nitrate concentrations are consistent with patterns reported across rural groundwater systems in West Africa, where natural aquifer characteristics, intensive agricultural activities, and inadequate sanitation infrastructure collectively contribute to nitrate enrichment [17,18]. These results highlight the vulnerability of shallow aquifers in the Guinea savanna zone to both natural and human-induced contamination, underscoring the need for targeted interventions, including source protection, pH correction, and nitrate-removal technologies, to safeguard public health.

### 4.6. Health Risk Implications of Nitrate-Nitrogen Exposure

The nitrate-nitrogen health risk assessment offers a more comprehensive evaluation of groundwater quality by quantifying population-level exposure risk, rather than relying solely on guideline compliance. In this study, nitrate contamination is identified as a potential non-carcinogenic health concern, particularly for vulnerable population groups. At a mean nitrate-nitrogen concentration of 23.28 mg/L, the hazard quotient (HQ) exceeded the 1 threshold for infants (HQ = 1.14), indicating a potential health risk under average exposure conditions. Although the HQ for children (HQ = 0.97) remained marginally below the threshold, it approached the level of concern, suggesting greater susceptibility than in adults and pregnant women. Under higher exposure scenarios, represented by the 95th percentile and maximum nitrate concentrations, HQ values increased substantially, with infants, children, adults, and pregnant women approaching or exceeding the acceptable risk threshold. Infants consistently exhibited the highest HQ values, attributable to their greater water intake relative to body weight, confirming their increased vulnerability to nitrate-related health effects, particularly methemoglobinemia. These findings align with established evidence that drinking-water nitrate limits are primarily intended to protect infants from methemoglobinemia, while other potential chronic health effects remain under investigation [6].

Spatial analysis reveals that elevated HQ values are primarily associated with nitrate concentrations in the North Gonja District, where both the frequency and magnitude exceed those observed in the North-East Gonja District. This pattern indicates that risk is concentrated in specific hydrogeological and land-use settings rather than uniformly distributed across the study area. The substantial variability in nitrate concentrations, as reflected by elevated upper-bound values and widespread exceedances of the WHO guideline value, suggests localized contamination hotspots linked to anthropogenic nitrogen inputs. Potential sources include leaching from on-site sanitation facilities such as pit latrines, agricultural fertilizer application, livestock waste, and settlement activities within groundwater recharge zones. Comparable patterns of nitrate enrichment associated with anthropogenic sources have been documented in groundwater systems in Ghana and other sub-Saharan African countries [15]. The co-occurrence of elevated nitrate concentrations and acidic groundwater conditions observed in this study further supports the hydrogeochemical plausibility of the identified risk patterns. Nitrification processes generate acidity, particularly in aquifers with limited buffering capacity, thereby enhancing nitrate mobility and persistence within groundwater systems. These hydrochemical interactions are widely recognized in groundwater studies, where nitrate contamination is influenced by both geochemical conditions and anthropogenic activities.

Consequently, the health risk assessment indicates that compliance with guideline values alone may underestimate actual exposure risk. Although the World Health Organization guideline value of 11.3 mg/L NO_3_-N is generally considered protective for most populations, the calculated HQ for infants approached the threshold of concern (HQ = 0.55) even at this concentration. This finding underscores the importance of considering population-specific exposure scenarios, particularly in rural communities where infants may consume untreated groundwater or infant formula prepared with contaminated water. Such precautionary approaches are strongly supported by international drinking-water guidelines, which emphasize risk-based management rather than exclusive reliance on compliance monitoring [1].

These findings highlight the need to integrate risk-based monitoring and intervention strategies into rural water supply management. Priority actions include targeted nitrate monitoring in high-risk communities, protection of groundwater recharge zones, improved sanitation planning to reduce nitrogen loading, and community-level risk communication. The implementation of Water Safety Plans (WSPs), which address both microbial and chemical hazards, is particularly relevant in these settings because it enables systematic identification, assessment, and control of risks throughout the water supply chain.

### 4.7. Relevance to Sustainable Development Goals (SDGs)

This study directly supports SDG 6.1 (universal access to safe and affordable drinking water) and SDG 3.9 (reducing deaths from hazardous chemicals and pollution). By providing disaggregated data for underserved rural districts, the study fills a critical gap in evidence needed for targeted intervention planning and resource allocation. It reinforces the call for safely managed water systems that are monitored, treated, and sustained through both infrastructure and behaviour change.

### 4.8. Study Strengths and Limitations

A principal strength of this study is its dual emphasis on microbial and chemical water quality at both source and household levels, offering a comprehensive perspective on contamination dynamics throughout the water supply chain. Nevertheless, the cross-sectional design restricts the ability to determine causal relationships between observed contamination patterns and potential risk factors. The study was conducted from January to March 2025, during the dry season, and therefore represents a dry-season assessment of drinking water quality. Consequently, seasonal variations in microbial contamination, nitrate transport and leaching, dilution effects, and recharge-driven hydrogeochemical processes were not fully addressed. Because groundwater quality in the Savannah Region is strongly influenced by rainfall patterns and recharge dynamics, these findings may not capture seasonal variability. Future research should include both dry- and rainy-season sampling to provide a more complete understanding of seasonal fluctuations and to enhance water-quality and health-risk assessments.

While this study recommends routine chlorination and residual chlorine monitoring as essential microbial risk-control measures, the sampled water systems were primarily groundwater-based boreholes and a limited number of mechanized systems, rather than surface water sources. The consistently low turbidity observed in source-water samples indicates minimal suspended particulate matter and a reduced likelihood of significant organic precursor loads compared to eutrophic or highly turbid surface waters. Consequently, the potential for chlorination by-product formation, such as trihalomethanes, is presumed to be lower in these systems. However, this assumption could not be directly verified because dissolved organic carbon, natural organic matter, and disinfection by-products were not analyzed. Additionally, pesticides, emerging contaminants, dissolved oxygen, and redox potential were not included within the analytical scope of this study. Therefore, the interpretation of nitrification–denitrification processes relied primarily on nitrate–nitrogen and pH patterns rather than direct redox measurements. Future research should incorporate indicators of organic matter, disinfection by-product monitoring, pesticides and emerging pollutants, dissolved oxygen, and redox potential, particularly in shallow boreholes, flood-prone areas, and agricultural recharge zones.

## 5. Conclusions and Recommendations

This study presents comprehensive evidence regarding drinking water quality challenges in the North Gonja and North-East Gonja Districts of the Savannah Region, Ghana. Groundwater sources in North-East Gonja generally demonstrated acceptable microbiological and chemical quality. In contrast, North Gonja exhibited substantially higher microbial contamination, elevated nitrate-nitrogen concentrations, acidic groundwater conditions, and a widespread absence of residual chlorine. Additionally, household-stored drinking water showed significantly higher microbial contamination than source water, indicating deterioration of water quality between collection and consumption. A health risk assessment indicated that nitrate exposure may pose non-carcinogenic health risks, particularly for infants and children.

These findings highlight significant public health concerns in rural northern Ghana. The widespread presence of total coliforms and Escherichia coli in household drinking water, together with elevated nitrate concentrations in several groundwater sources, suggests that communities are exposed to both acute and chronic health risks. The pronounced differences between North Gonja and North-East Gonja further illustrate the influence of local hydrogeological conditions, land-use practices, and water management systems on drinking water safety. These results emphasize the need to integrate microbiological monitoring, chemical surveillance, and health risk assessment into routine drinking water management programs.

To enhance drinking water safety and protect public health, priority should be given to strengthening Water Safety Planning, routine water quality monitoring, source protection, and community-based chlorination programs. Interventions should also promote safe household water handling, storage, and point-of-use treatment to reduce post-collection contamination. In areas with elevated nitrate concentrations, targeted surveillance and mitigation measures should be implemented, particularly to safeguard vulnerable groups such as infants and children. Collectively, these actions will contribute to safer drinking water supplies and advance progress toward Sustainable Development Goal 6 in rural Ghana.

## Figures and Tables

**Table 1:** Analytical methods for water quality parameters.

Parameter	Analytical Method
pH	Measured using a calibrated benchtop pH meter (Method SM 4500-H^+^ B)
Electrical Conductivity (EC)	Determined using a conductivity meter (Method SM 2510 B)
Turbidity	Assessed using a nephelometric turbidity meter (Method SM 2130 B)
Nitrate (NO_3_^−^N)	SM 4500-NO_3_ D (Ion Selective Electrode)
Fluoride (F^−^)	SM 4500-F^−^ C (Ion Selective Electrode)
Chloride (Cl^−^)	EPA 9212 (Ion Selective Electrode)
Trace metals	Analysed by Graphite Furnace Atomic Absorption Spectroscopy (GFAAS) following digestion (Method SM 3113 B)

**Table 2:** (A). Descriptive statistics of physical and operational water quality parameters of source water (*n* = 112). (B). Descriptive statistics of major ions and general chemical parameters of source water (*n* = 112). (C). Descriptive statistics of trace metal concentrations in groundwater sources from North Gonja and North-East Gonja Districts, Ghana (*n* = 112).

(**A**)								
**Parameter**	**Unit**	***n***	**Mean**	**SD**	**Min**	**Max**	**Guideline Value**	**Exceedance *n* (%)**
Residual chlorine	mg/L	112	0.00	0.00	0.00	0.00	0.2–0.5	112 (100.0)
pH	pH unit	112	6.25	1.06	4.40	8.52	6.5–8.5	75 (67.0)
Conductivity	µS/cm	112	225.93	266.22	13.00	1056.00	No health-based guideline	0 (0.0)
TDS	mg/L	112	146.86	173.04	8.45	686.40	≤1000	0 (0.0)
Turbidity	NTU	112	0.67	2.69	0.02	26.30	≤5	3 (2.7)
Alkalinity	mg CaCO_3_/L	112	2.25	3.47	0.05	14.24	No health-based guideline	0 (0.0)
(**B**)								
**Parameter**	**Unit**	***n***	**Mean**	**SD**	**Min**	**Max**	**Guideline Value**	**Exceedance *n* (%)**
Fluoride	mg/L	112	0.14	0.21	0.01	0.98	≤1.5	0 (0.0)
Chloride	mg/L	112	10.59	23.48	0.72	206.67	≤250	0 (0.0)
Nitrate-N	mg/L	112	23.28	23.27	0.35	72.00	≤11.3	55 (49.1)
Sulfate	mg/L	112	10.86	17.18	0.38	133.82	≤250	0 (0.0)
(**C**)								
**Parameter**	**Unit**	***n***	**Mean**	**SD**	**Min**	**Max**	**Guideline Value**	**Exceedance *n* (%)**
Arsenic	mg/L	112	0.00	0.00	0.00	0.00	≤0.01	0 (0.0)
Cadmium	mg/L	112	0.00	0.00	0.00	0.002	≤0.003	0 (0.0)
Chromium	mg/L	112	0.00	0.00	0.00	0.00	≤0.05	0 (0.0)
Copper	mg/L	112	0.004	0.014	0.000	0.087	≤2.0	0 (0.0)
Iron	mg/L	112	0.052	0.045	0.000	0.266	≤0.3	0 (0.0)
Manganese	mg/L	112	0.000	0.000	0.000	0.000	≤0.4	0 (0.0)
Lead	mg/L	112	0.000	0.000	0.000	0.000	≤0.01	0 (0.0)

**Table 3:** Non-carcinogenic health risk (HQ) for nitrate-nitrogen exposure across population groups.

Exposure Scenario	NO_3_-N (mg/L)	Infants (HQ)	Children (HQ)	Adults (HQ)	Pregnant Women (HQ)
Mean concentration	23.28	1.14	0.97	0.42	0.48
95th percentile	64.78	3.16	2.70	1.16	1.33
Maximum concentration	72.00	3.51	3.00	1.29	1.48
WHO guideline value	11.30	0.55	0.47	0.20	0.23

**Table 4:** Chi-Square results.

Parameter	χ^2^ Value	Df	*p*-Value
Nitrate (NO_3_-N)	13.27	1	<0.05
pH (<6.5)	25.69	1	<0.05
Turbidity (>5 NTU)	0.00	1	>0.05

## Data Availability

The datasets generated and analysed during the current study are available from the corresponding author on reasonable request.
